# Monocyte to high-density lipoprotein cholesterol ratio as long-term prognostic marker in patients with coronary artery disease undergoing percutaneous coronary intervention

**DOI:** 10.1186/s12944-019-1116-2

**Published:** 2019-10-22

**Authors:** Ting-Ting Wu, Ying-Ying Zheng, You Chen, Zi-Xiang Yu, Yi-Tong Ma, Xiang Xie

**Affiliations:** 1 Department of Cardiology, First Affiliated Hospital of Xinjiang Medical University, Urumqi, 830054 People’s Republic of China; 2 Department of Cardiology, First Affiliated Hospital of Zhengzhou University, Zhengzhou, 450052 People’s Republic of China

**Keywords:** Monocyte to high-density lipoprotein cholesterol ratio, Prognostic markers, Coronary artery disease, Percutaneous coronary intervention

## Abstract

**Background:**

The relation between monocyte to high-density lipoprotein cholesterol ratio (MHR) and coronary artery disease (CAD) undergoing percutaneous coronary intervention (PCI) remains controversial. The present study aims to assess the prognostic value of MHR in patients with CAD who underwent PCI.

**Methods:**

A total of 673 CAD patients were retrospectively enrolled and divided into four groups according to MHR values. Multivariate Cox regression analysis was performed to study the effects of different variables to clinical outcomes reported as major adverse cardiac events (MACE) and all-cause mortality (ACM).

**Results:**

In a multivariate Cox analysis, after adjustment of other confounders, MHR was found to be an independent predictor of ACM (HR: 3.655; 95% CI: 1.170–11.419, *P* = 0.026) and MACE (HR =2.390, 95% CI 1.379–4.143, *p* < 0.002). Having a MHR in the third and fourth quartile were associated with a 2.83-fold and 3.26 -flod increased risk of MACE.

**Conclusions:**

MHR is an independent predictor of ACM and MACE in CAD patients undergoing PCI.

## Introduction

During the past three decades, percutaneous coronary intervention (PCI) has become one of the dominant methods for revascularization in patient with coronary artery disease (CAD). Previous study suggested that inflammation, oxidative stress, and endothelial dysfunction play important roles in the initiation and progression of atherosclerotic process [1]. Mounting interest focuses on the identification of new prognostic markers better enabling the category of patients who are at higher risk for future cardiovascular events. Humoral biomarkers of inflammation are correlated with initiation, progression, destabilization of an atherosclerotic plaque and appear to correlate with future cardiovascular events warranting investigation of more specific associations [2, 3].

Circulating monocytes as a source of various cytokines and molecules, interact with platelets and endothelial cells and leading to aggravation of inflammatory, pro-thrombotic pathways [4, 5]. High-density lipoprotein cholesterol (HDL-C) defuse these pro-inflammatory and pro-oxidant effects of monocytes by inhibiting the migration of macrophages and oxidation of the low-density lipoprotein cholesterol (LDL-C) molecules as well as promoting the efflux of cholesterol from these cells [6, 7]. Because of this, properties such as monocyte count to HDL-C ratio (MHR) could show the inflammatory status of a patient. Consistent with this, the association between increased MHR and cases of atherosclerosis has been demonstrated and MHR has emerged as a new cardiovascular prognostic marker in previous studies [8–11]. In the present study, we aimed to investigate the relationship between MHR and the clinical outcomes of CAD patients after PCI.

## Methods

### Study population

**Fig. 1:**
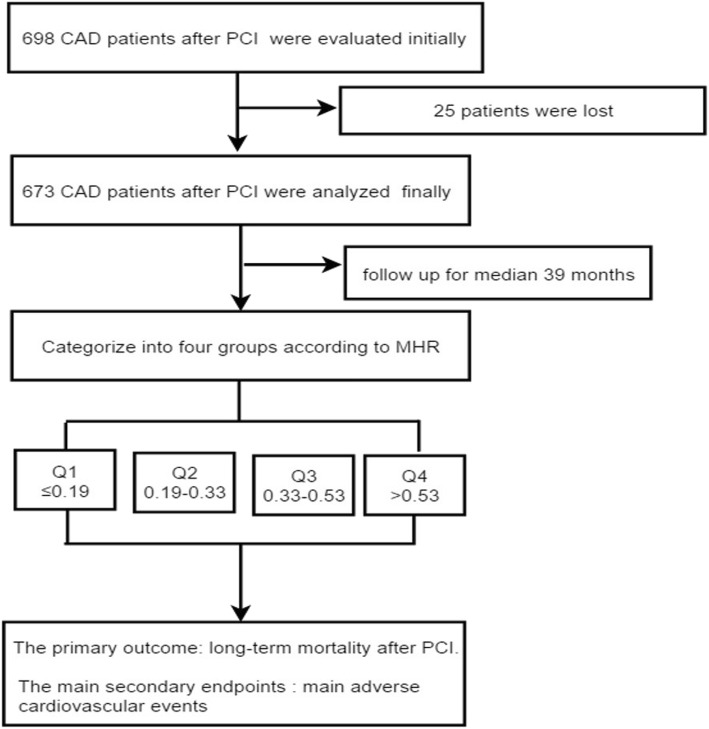
The follow chart of participants inclusion

We performed a 10-year retrospective cohort study, from January 2008 to December 2016 in the First Affiliated Hospital of Xinjiang Medical University, according to the strict inclusion criteria. Patients with serious heart failure, rheumatic heart disease, valvular heart disease, congenital heart disease and pulmonary heart disease were exclude. We also excluded patients with end-stage renal disease and serious dysfunction of the liver, clinical evidence of cancer, active or chronic inflammatory or autoimmune diseases, active infection and patients who received blood transfusion recently. CAD was defined as the presence of at least one significant coronary artery stenosis of ≥50% luminal diameter on coronary angiography. In the initial,we enrolled 698 consecutive patients with a diagnosis of CAD who underwent PCI. During the follow-up period, 25 patients were lost (changing telephone number or moving to another place). 673 patients were enrolled finally. The study complied with the Declaration of Helsinki, and the protocol was approved by the Human Ethical Committee of the First Affiliated Hospital of Xinjiang Medical University. Because of the retrospective design of the study, the need to obtain informed consent from eligible patients was waived by the ethics committee. Major adverse cardiac events (MACE) and all-cause mortality (ACM) were reported as the clinical outcomes. Follow-up data were obtained by review of the medical records and/or telephone interview with the patient or family members.A flowchart outlining our study was shown in Fig. 1**.**

### Definitions

Hypertension was defined as a systolic blood pressure of ≥140 mmHg and/or a diastolic blood pressure of ≥90 mmHg in at least 2 measurements or use of any antihypertensive drug. Diabetes mellitus was defined as fasting plasma glucose level ≥ 7.1 mmol/L on multiple measurements or current use of anti-diabetic medications. Hypercholesterolemia was considered as total serum cholesterol of ≥200 mg/dL or the use of lipid-lowering medications. Family history of CAD was considered in case of history of CAD or sudden cardiac death in a first-degree relative before the age of 55 years for men and 65 years for women. Smoking and drinking status was defined as current tobacco and alcohol use. Cardiovascular mortality, re-hospitalization due to unstable or progressive angina, heart failure, stroke, re-infarction, re-stents implanted, and coronary artery bypass grafting were regarded as MACE. Scoring of severity of coronary artery disease was performed with a modification of the coronary atherosclerosis scoring system which was called the standard of Gensini Method.

### Clinical and demographic characteristics

Peripheral venous blood samples of the patients were obtained on admission to the inpatient ward. Data regarding clinical and demographic characteristics including age, sex, history of hypertension and diabetes mellitus, smoking status, alcohol intake, family history of coronary artery disease and medications were collected from medical records. Left ventricular ejection fraction and laboratory data including urea and creatinine levels, total cholesterol(TC), LDL-C,HDL-C and triglycerides(TG) levels, complete blood cell count and white blood cell (WBC) subgroups count were noted. During hospitalization and follow-up period, β-blocker, angiotensin-converting enzyme inhibitor, and statin were administered to all patients unless contraindicated.

### Statistical analysis

All analyses were performed using SPSS 22.0 for Windows statistical software (SPSS Inc., Chicago, IL, USA).The normality of the distribution of continuous variables was evaluated by the Kolmogorov-Smirnov test. Continuous variables were expressed as Mean ± standard deviation. Parametric patient characteristics were compared using one-way ANOVA, whereas non-parametric characteristics were compared using the Kruskal–Wallis test. Categorical variables were summarised as percentages and compared using the Chi-square (x^2^) test. Stepwise regression was used to deal with the collinear problem. Multivariate Cox proportional hazard model were used for determination of independent parameters for MACE and ACM. To construct the Cox model, univariate models for each of the all predictor variables were run, with those variables that were significant (*P* < 0.05) in univariate Cox models were then simultaneously entered into a multivariable Cox model. The Hazard ratios (HRs) and 95% confidence intervals (CIs) were calculated. The cumulative survival curve for MACE and ACM was constructed using the Kaplan-Meier method and compared using the log-rank test. *P* < 0.05 was considered significant. Receiver operating characteristic (ROC) curve was performed to discuss the diagnostic value of risk factors for the prediction of poor prognisis.

## Results

A total of 673 patients (543 male, 80.7%; mean age 59.1 ± 10.8) with the diagnosis of CAD after PCI were enrolled. The mean follow-up period was 39 ± 25 months. The clinical, echocardiographic, and laboratory data of the study population are given in Table 1**.**

**Table 1:** Clinical and laboratory characteristics

Parameters	Total(*N* = 673)
Age (years)	59.1 ± 10.8
Male sex [n (%)]	543(80.7)
Smoking [n (%)]	315(46.8)
Alcohol intake[n (%)]	251(37.3)
Hypertension [n (%)]	323(48.0)
Family history of CAD [n (%)]	145(21.5)
Hypercholesterolemia [n (%)]	287(42.6)
Diabetes [n (%)]	170(25.3)
LVEF (%)	61 ± 7
Regular drug taking[n (%)]	594(88.3)
Pre-procedural laboratory parameters	
SBP (mmHg)	134 ± 26
HR (bpm)	75 ± 10
WBC (× 10^9^/L)	7.49 ± 2.33
Monocyte (× 10^9^/L)	0.52 ± 0.22
TG(mmol/l)	1.61(1.18–2.48)
TC(mmol/l)	4.04 ± 1.21
LDL-C(mmol/l)	1.32(1.06–2.22)
HDL-C(mmol/l)	1.48(0.93–2.48)
Gensini score	36(20–64)
MHR	0.40 ± 0.30
MHR quartiles	
Q1(≤0.19)	167(24.8)
Q2(0.19–0.33)	177(26.3)
Q3(0.33–0.53)	158(23.5)
Q4(> 0.53)	171(25.4)
Follow-up time (months)	39 ± 25

*WBC* White blood cell, *SBP* Systolic blood pressure, *HR* Heart ratio, *TG* Triglyceride, *TC* Cholesterol, *HDL-C* High-density lipoprotein cholesterol, *LDL-C* Low-density lipoprotein cholesterol, *LVEF* Left ventricular ejection fraction; Regular drug taking: angiotensin-converting enzyme inhibitor, angiotensin II receptor blocker and statin were administered by medical advice, *MHR* Monocyte/HDL-C ratio

### Comparison of the quartile groups

The study population were assigned into quartiles (Q) based on pre-ablation MHR (Q1:≤ 0.19; Q2: 0.19–0.33; Q3: 0.33–0.53; Q4: > 0.53). Baseline characteristics, laboratory parameters, and coronary angiographic findings of the patient groups according to the MHR quartile groups are presented in Table 2. Older age, higher rate of male, higher SBP, WBC counts, monocyte counts, fasting blood glucose(FBG), LDL-C, and lower HDL-C were more prevalent in the high MHR level group (*P* < 0.05).

**Table 2:** Clinical and laboratory characteristics according to the monocyte-to-HDL ratio quartiles

Parameters	Q1(≤0.19) *N* = 167	Q2(0.19–0.33) *N* = 177	Q3(0.33–0.53) *N* = 158	Q4(> 0.53) *N* = 171	*P*
Age(years)	57 ± 11	58 ± 11	60 ± 11	59 ± 11	0.045
Male sex [n (%)]	120(71.9%)	144(81.8%)	130(82.3%)	149(87.3%)	0.004
Smoking [n (%)]	69(41.3%)	84(47.5%)	82(51.9%)	80(46.8%)	0.297
Alcohol intake [n (%)]	54(32.3%)	62(35.0%)	69(43.7%)	66(38.6%)	0.171
Hypertension [n (%)]	74(44.3%)	82(46.3%)	80(50.6%)	87(50.9%)	0.551
Family history of CAD [*n* (%)]	34(20.4%)	38(21.5%)	34(21.5%)	39(22.8%)	0.960
Hypercholesterolemia[n (%)]	65(38.9%)	70(39.5%)	76(48.1%)	76(44.4%)	0.286
Diabetes [n (%)]	41(24.6%)	44(24.9%)	40(25.3%)	45(26.3%)	0.984
LVEF (%)	61 ± 7	62 ± 7	61 ± 7	61 ± 8	0.401
Gensini score	43 ± 43	45 ± 42	46 ± 37	52 ± 35	0.150
Laboratory parameters					
SBP (mmHg)	130 ± 21	131 ± 24	135 ± 29	139 ± 29	0.003
HR (bpm)	73 ± 9	74 ± 10	75 ± 11	76 ± 10	0.068
WBC(×10^9^/l)	6.73 ± 2.14	7.36 ± 1.88	7.58 ± 2.43	8.28 ± 2.58	< 0.001
MO(×10^9^/l)	0.36 ± 0.05	0.51 ± 0.18	0.54 ± 0.22	0.67 ± 0.23	< 0.001
HB(× 10^12^/l)	141 ± 14	140 ± 16	139 ± 15	139 ± 14	0.499
RDW(%)	13.21 ± 0.98	13.17 ± 0.79	13.15 ± 0.81	13.24 ± 0.20	0.778
PLT(×10^9^/l)	212 ± 59	217 ± 72	211 ± 61	212 ± 61	0.847
BUN(mmol/l)	5.49 ± 1.41	5.53 ± 1.63	5.40 ± 1.66	5.40 ± 1.70	0.823
FBG(mmol/l)	6.15 ± 2.55	6.27 ± 2.51	6.54 ± 3.03	6.93 ± 2.91	0.045
TG(mmol/l)	2.12 ± 1.25	1.87 ± 1.23	1.87 ± 1.15	2.37 ± 1.70	0.001
TC(mmol/l)	4.53 ± 1.06	3.97 ± 1.09	3.93 ± 1.46	3.73 ± 1.07	< 0.001
HDL-C(mmol/l)	2.89 ± 0.95	2.05 ± 0.78	1.31 ± 0.51	0.86 ± 0.30	< 0.001
LDL-C(mmol/l)	1.27 ± 0.50	1.51 ± 0.98	1.97 ± 1.10	2.14 ± 0.97	< 0.001
MHR	0.12 ± 0.05	0.25 ± 0.04	0.42 ± 0.06	0.82 ± 0.31	< 0.001
IBIl(umol/l)	8.57 ± 4.43	9.21 ± 5.52	8.89 ± 5.22	8.26 ± 4.56	0.318
DBIL(umol/l)	3.17 ± 2.02	3.51 ± 2.28	3.45 ± 2.59	3.10 ± 2.34	0.270
Drugs parameters					
Regular drugs taking	142(85%)	153(86.4%)	143(90.5%)	156(91.2%)	0.215
Clinical result					
Follow up time(m)	45 ± 26	45 ± 29	37 ± 25	31 ± 16	< 0.001
ACM [*n* (%)]	1(0.6%)	1(0.6%)	4(2.5%)	8(4.7%)	0.022
MACE [*n* (%)]	15(9%)	26(14.7%)	28(17.7%)	33(19.3%)	0.044

*Mo* Monocyte, *PLT* Platelet, *BUN* Blood urea nitrogen, *HB* Hemoglobin B, *FBG* Fasting blood glucose, *RDW* Red cell distribution width, *IBil* Indirect bilirubin, *DBIL* Direct bilirubin, *ACM* All-cause mortality, *MACE* Major adverse cardiac events

### Comparison of clinical outcomes

During long term follow-up, the prevalence of ACM and MACE occurred more frequently in the fourth quartile group. The results of Cox regression analysis for long-term clinical outcomes are also shown in Table 3-4**.** According to univariate Cox proportional hazard regression analysis, age, MHR, left ventricular ejection fraction (LVEF), diabetes, HR (heart ratio), haemoglobin B (HB), direct bilirubin DBIL, LDL-C and Gensini score were significantly associated with ACM (*P* < 0.05) **(**Table 3). Multivariate Cox proportional hazard regression analysis showed that age.

**Table 3:** Univariate and multivariate Cox proportional Hazard modeling results of ACM

Variables	Univariate model			Multivariate model		
	HR	95%CI	*P*	HR	95%CI	*P*
Age	1.097	1.035–1.163	0.002	1.071	1.005–1.141	0.035
Male sex	1.792	0.558–5.759	0.328	–	–	–
Smoking	0.812	0.281–2.344	0.7	–	–	–
Dringking	0.644	0.201–2.067	0.46	–	–	–
Hypertension	2.627	0.822–8.398	0.103	–	–	–
Family history of CAD	1.114	0.310–4.009	0.868	–	–	–
Hypercholesterolemia	1.018	0.352–2.946	0.974	–	–	–
Diabetes	3.488	1.206–10.093	0.021	3.038	0.856–10.783	0.086
LVEF (%)	0.929	0.885–0.976	0.004	0.958	0.899–1.021	0.190
HR	1.052	1.00–1.097	0.017	1.061	1.005–1.120	0.031
WBC	1.123	0.949–1.329	0.177	–	–	–
HB	0.95	0.919–0.983	0.003	0.966	0.928–1.007	0.101
RDW	1.37	0.871–2.155	0.173	–	–	–
PLT	0.999	0.991–1.007	0.816	–	–	–
BUN	1.187	0.903–1.561	0.22	–	–	–
FBG	1.084	0.934–1.257	0.288	–	–	–
TG	1.017	0.687–1.505	0.932	–	–	–
TC	1.117	0.747–1.670	0.59	–	–	–
LDL-C	1.715	1.248–2.356	0.001	2.094	1.400–3.134	< 0.001
DBIL	1.217	1.057–1.400	0.006	1.267	1.078–1.469	0.004
IBIL	0.997	0.897–1.108	0.956	–	–	–
Gensini score	1.013	1.007–1.019	< 0.001	1.019	1.007–1.032	0.002
MHR	5.082	1.957–13.196	0.001	3.655	1.170–11.419	0.026

**Table 4:** Univariate and multivariate Cox proportional Hazard modeling results of MACE

Variables	Univariate model			Multivariate model		
	HR	95%CI	*P*	HR	95%CI	*P*
Age	1.018	1.000–1.037	0.049	1.005	0.985–1.026	0.618
Male sex	1.678	1.083–2.599	0.02	1.281	0.768–2.138	0.343
Smoking	1.113	0.754–1.642	0.59	–	–	–
Drinking	1.185	0.798–1.760	0.4	–	–	–
Hypertension	1.528	1.025–2.277	0.037	1.551	0.996–2.416	0.052
Family history	1.536	0.975–2.420	0.064	–	–	–
Hypercholesterolemia	1.244	0.842–1.838	0.273	–	–	–
Diabetes	1.919	1.295–2.843	0.001	1.911	1.241–2.941	0.003
LVEF (%)	0.989	0.967–1.011	0.32	–	–	–
HR	1.024	1.006–1.042	0.01	1.021	1.001–1.041	0.042
WBC	1.076	1.000–1.156	0.049	1.010	0.930–1.097	0.812
HB	0.978	0.965–0.991	0.001	0.980	0.965–0.995	0.008
RDW	1.134	0.941–1.366	0.187	–	–	–
PLT	1	0.997–1.002	0.791	–	–	–
BUN	1.077	0.966–1.200	0.18	–	–	–
FBG	1.087	1.027–1.150	0.004	0.998	0.934–1.067	0.958
TG	0.979	0.837–1.146	0.795	–	–	–
TC	0.993	0.842–1.172	0.937	–	–	–
LDL-C	1.428	1.230–1.657	< 0.001	1.411	1.178–1.690	< 0.001
DBIL	1.062	0.980–1.150	0.143	–	–	–
IBIL	0.972	0.932–1.012	0.171	–	–	–
Gensini score	1.006	1.001–1.011	0.022	1.006	1.001–1.011	0.029
MHR	3.414	2.148–5.427	< 0.001	2.390	1.379–4.143	0.002
MHR quartiles						
Q1	Ref	Ref	–	Ref	Ref	–
Q2	1.576	0.833–2.982	0.162	1.652	0.844–3.233	0.143
Q3	2.654	1.414–4.981	0.002	2.831	1.433–5.596	0.003
Q4	4.357	2.344–8.099	< 0.001	3.258	1.604–6.619	0.001

(HR: 1.071; 95% CI: 1.005–1.141, *P* = 0.035), heart ratio (HR: 1.061; 95% CI: 1.005–1.120, *P* = 0.031), DBIL (HR: 1.267; 95% CI: 1.078–1.469, *P* = 0.004), LDL-C(HR: 2.094; 95% CI: 1.400–3.134, *P* < 0.001) Gensini sore (HR: 1.019; 95% CI: 1.007–1.032, *P* = 0.002) and MHR (HR: 3.655; 95% CI: 1.170–11.419, *P* = 0.026) were independent predictors of ACM after adjustment of other variables (Table 3).

As shown in Table 4, univariate Cox proportional hazard regression analysis showed age, MHR, male, diabetes, hypertension, HR, HB, FBG, WBC, LDL-C, Gensini score were significantly associated with MACE (*P* < 0.05). In multivariate Cox proportional hazard regression analysis, MHR was also found as an independent predictor of MACE (HR =2.390, 95% CI 1.379–4.143, *p* < 0.002), along with diabetes mellitus (HR = 1.911, 95% CI 1.241–2.941, *p* = 0.003), hypertension (HR = 1.576, 95% CI 1.011–2.455, *p* = 0.044), hemoglobin (HR = 0.980, 95% CI 0.965–0.995, *p* = 0.008) heart ratio (HR = 1.021, 95% CI 1.001–1.041, *p* = 0.042), LDL-C(HR:1.411; 95% CI: 1.178–1.690, *P* < 0.001), and Gensini score (HR = 1.006, 95% CI 1.001–1.011, *p* = 0.029) were found as independent predictors of MACE. Having a MHR in the third and fourth quartile were associated with a 2.83-fold (HR=2.831, 95% CI 1.433–5.596, *p* = 0.003) and 3.26-flod (HR=3.258, 95% CI 1.604–6.619, *p* = 0.001) increased risk of MACE.

**Fig. 2:**
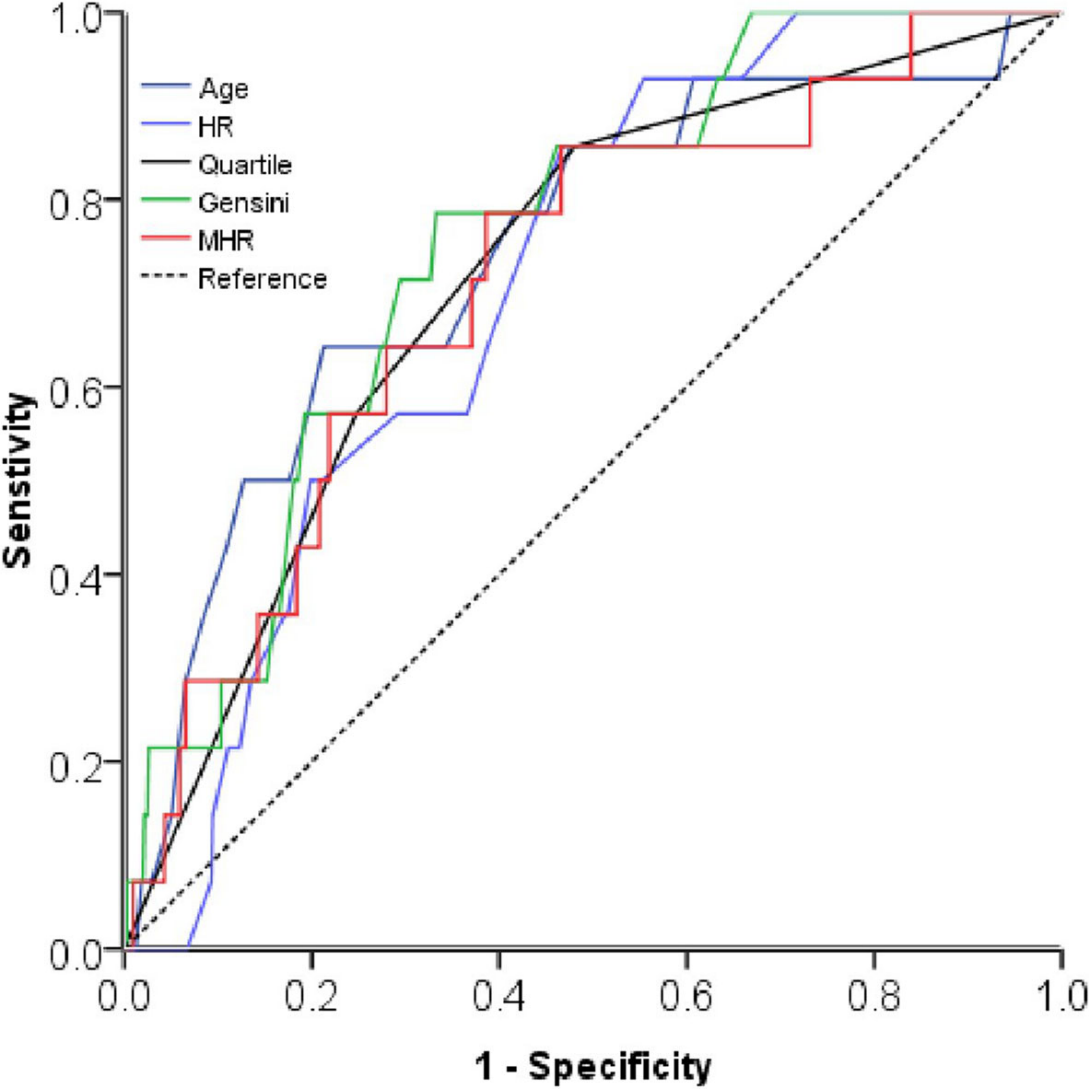
Receiver operating curve (ROC) for the analysis of possible predictors of all-cause mortality in the study population. AUC indicates area under curve

**Fig. 3:**
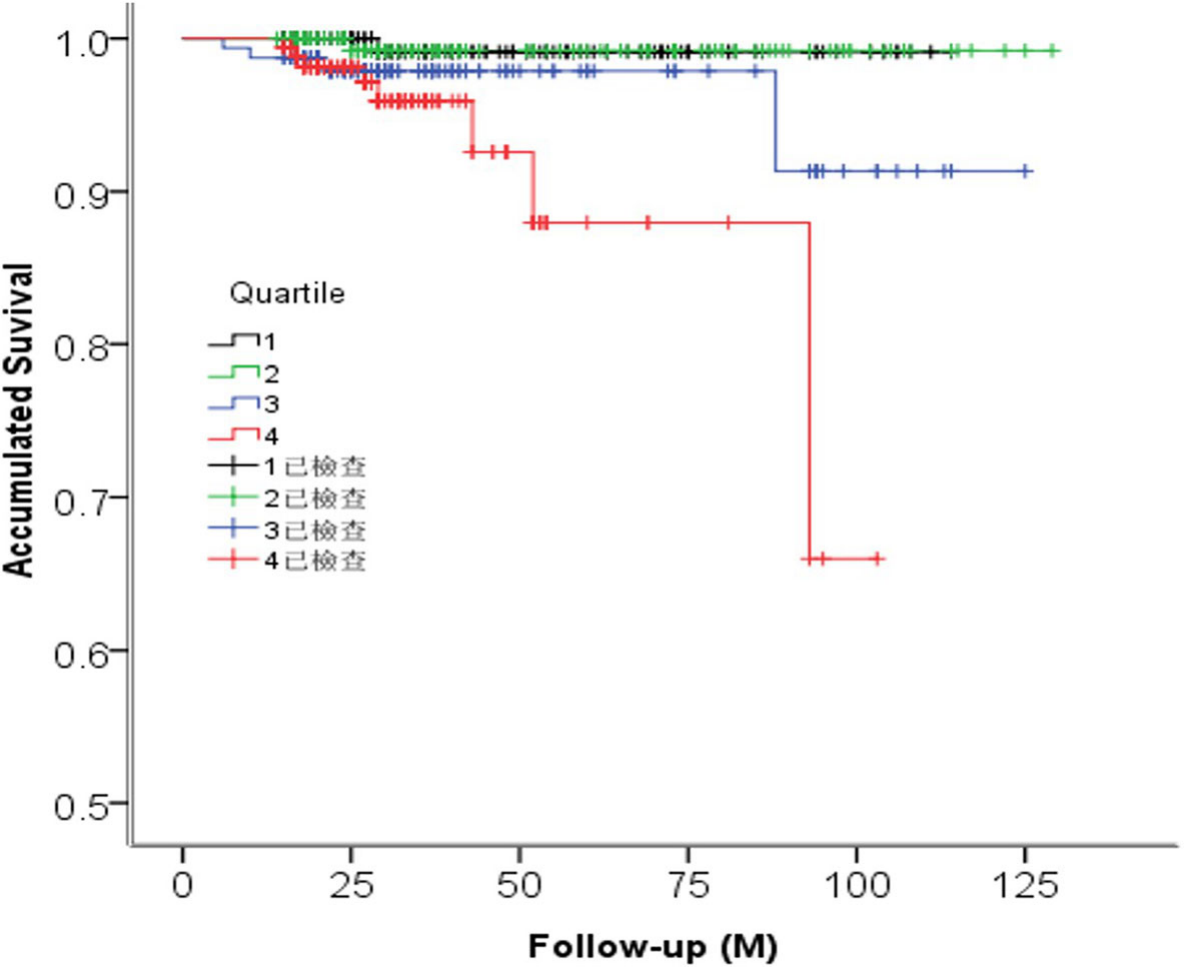
The Kaplan-Meier survival analysis for all-cause mortality

**Fig. 4:**
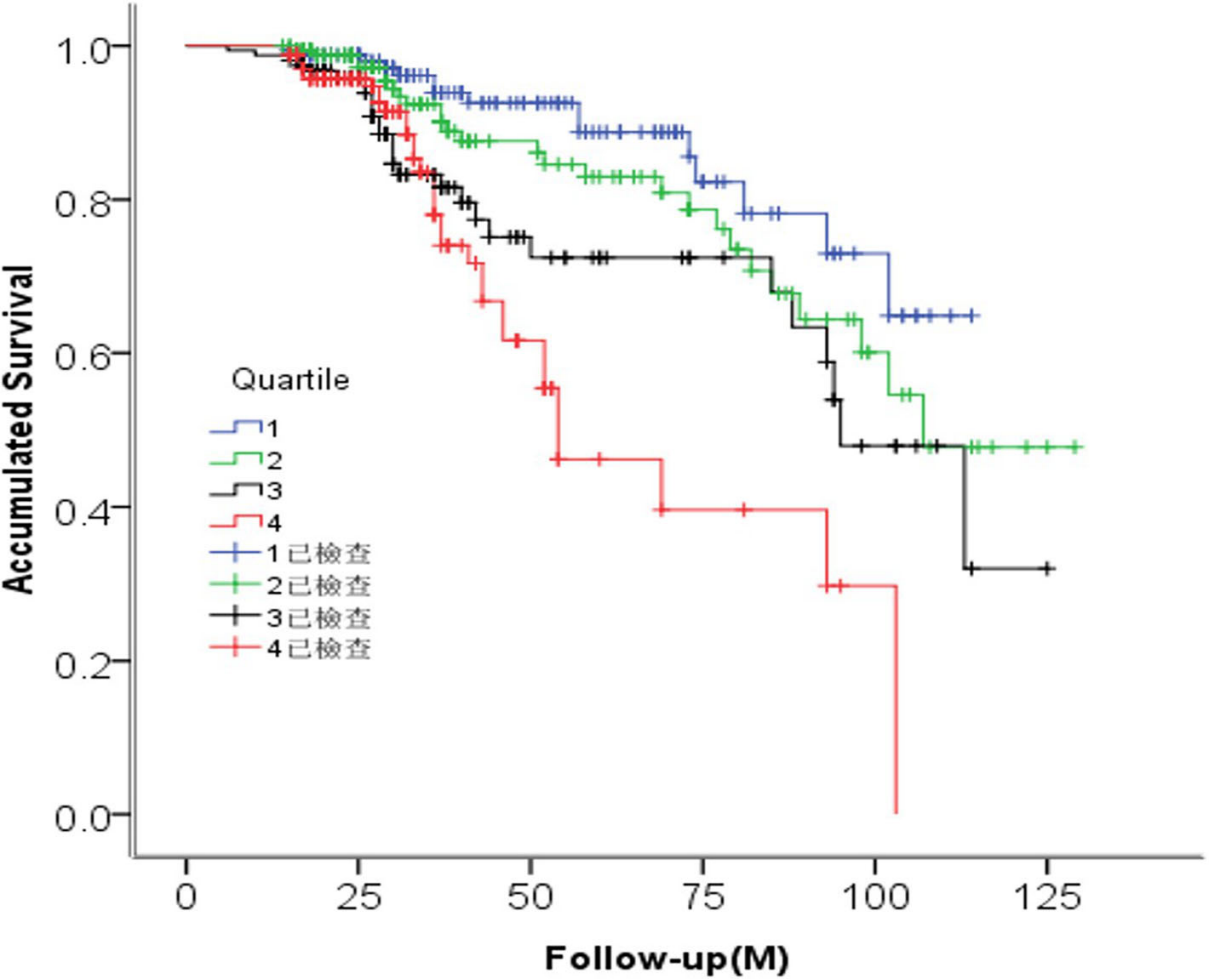
The Kaplan-Meier survival analysis for major adverse cardiovascular events

Receiver operating curve (ROC) analysis showed that MHR levels could predict ACM with a sensitivity of 78.6% and a specificity of 61.5% (AUC = 0.714, *p* = 0.006) (Fig. 2). The area under the curve (AUC) of age (AUC = 0.742), heart ratio (AUC =0.710), Gensini score (AUC =0.754), MHR quartile groups (AUC = 0.719) were all significant(*p* < 0.05). Kaplan-Meier curves among quartiles for both ACM and MACE (log-rank, *P* < 0.05), which represent worse outcomes as MHR increases, were shown in Fig. 3-4**.**

## Discussion

In our study, we found that elevated MHR is a useful marker of poor prognosis in patients with CAD undergoing PCI. In multivariate analysis, the MHR emerged as an independent predictor of ACM and MACE.

Recently, MHR is considered as a novel and surrogate marker of inflammatory status. Acikgoz et al. [12] reported a novel easily available inflammatory marker-MHR. In their study, the authors found an association between higher MHR and impaired endothelial function and systemic inflammation in patients with Behçet disease.. Previous studies also showed that MHR might a predictor for obstructive sleep apnea syndrome [13, 14]. You et al. [15] reported that there was a significant correlation between MHR and 3-month outcomes in patients with acute intracerebral hemorrhage. A significant relationship between blood cadmium and monocyte count and MHR among male fire officers was reported [16]. Cagli et al. [17] also reported an association of MHR with the abdominal aortic aneurysm size. Koçak et al. [18] demonstrated that the MHR is not an independent risk factor in idiopathic sudden hearing loss patients. However, high MHR values may be correlated with a poor prognosis. Furthermore, numerous researches about the relationship between MHR and cardiovascular disease have been reported recently. There is a relationship between the elevation of MHR and presence and severity of isolated coronary artery ectasia [19], asymptomatic organ damage in patients with primary hypertension [20], slow coronary flow [21], cardiac syndrome X [22], metabolic syndrome [23], infective endocarditi [24] and saphenous vein graft disease in coronary bypass [25]. It also has been reported to be related to cardiovascular outcomes in patients with chronic kidney disease [26], arterial fibrillation recurrence after cryoballoon based catheter ablation [27], mortality and arterial fibrillation recurrence after coronary artery bypass grafting [28], and contrast induced nephropathy in acute ST-segment elevation myocardial infarction (STEMI) patients treated with primary PCI [29]. Moreover, there are some researches about the recent evidence suggested that in patients with STEMI, the admission MHR values were independently correlated with poor prognosis as major adverse cardiovascular events and mortality [30–32]. Besides procedural problems related to PCI, up to now, several studies have consistently demonstrated that MHR is a reliable factor for inflammation and anti-oxidation and is associated with stent thrombosis in STEMI patients [33–37].

The exact mechanisms underlying the association between MHR and clinical outcomes after PCI are unknown. As previous studies have shown, atherosclerosis activates the adhesion molecule of endothelium cells and binds to mononuclear cells. When monocytes are bound to the arterial endothelium, they enter the endothelial lining and enter the intima to form foam cells. Activated monocytes interact with damaged or activated endothelium, affect lesional macrophage accumulation and plaque macrophage content [38], which lead to overexpression of some pro–inflammatory mediator molecules including monocyte chemotactic protein 1 ligand, vascular cell adhesion molecule 1, and intercellular adhesion molecule 1. Thereafter, monocytes differentiate into the macrophages that eventually ingest oxidized LDL-C and form the initial foamy cells [39]. The HDL-C molecules counteract macrophage migration and remove cholesterol debris from those cells. HDL-C suppresses hematopoietic stem cells and multipotent progenitor cell proliferation in the hematopoietic system [40]. HDL-C inhibits monocyte, leading to mechanisms that act as inhibit activated monocyte adhesion, spreading, and controlling the proliferation of progenitor cells that differentiate to monocytes [41, 42]. Besides its anti-inflammatory and anti-oxidative effects, HDL also promotes vasodilatation by increasing the expression of endothelial nitric oxide synthase [43].Therefore, monocytes exert a pro-inflammatory and pro-oxidant effects, but HDL-C functions as a reversal factor during these processes.

### Study limitations

Nonetheless, there are some limitations of this study. Firstly, the present study is retrospectively designed and is a single-center experience study. Secondly, a relatively small sample size is another limitation. Third, additional inflammation and thrombosis markers such as CRP, fibrinogen, plasma coagulation factors, or erythrocyte sedimentation rate were not evaluated in the present study.

## Conclusion

MHR can be calculated from simple blood analysis and could make a adverse prognostic value in CAD patients undergoing PCI.

## Data Availability

Due to confidentiality policies, data will not be shared.

## Abbreviations

CAD: Coronary artery disease
CAG: Coronary angiography
DM: Diabetes mellitus
EH: Essential hypertension
LVEF: Left ventricular ejection fraction
MHR: Monocyte to high-density lipoprotein cholesterol ratio

## References

[1] Hansson GK, Libby P, Tabas I (2015). Inflammation and plaque vulnerability. J Intern Med J Intern Med.

[2] Weber C, Noels H (2011). Atherosclerosis: current pathogenesis and therapeutic options. Nat Med.

[3] Libby P (2012). Inflammation in atherosclerosis. Arterioscler Thromb Vasc Biol.

[4] Hristov M, Heine GH (2015). Monocyte subsets in atherosclerosis. Hamostaseologie.

[5] Ancuta P, Wang J, Gabuzda D (2006). CD16+ monocytes produce IL-6, CCL2, and matrix metalloproteinase-9 upon interaction with CX3CL1-expressing endothelial cells. J Leukoc Biol.

[6] Tardif JC, Grégoire J, L'Allier PL, Ibrahim R, Lespérance J, Heinonen TM, Kouz S, Berry C, Basser R, Lavoie MA, Guertin MC (2007). Rodés-Cabau J; effect of rHDL on atherosclerosis-safety and efficacy (ERASE) investigators. Effects of reconstituted high-density lipoprotein infusions on coronary atherosclerosis: a randomized controlled trial. JAMA.

[7] Murphy AJ, Woollard KJ, Hoang A, Mukhamedova N, Stirzaker RA, McCormick SP, Remaley AT, Sviridov D, Chin-Dusting J (2008). High-density lipoprotein reduces the human monocyte inflammatory response. Arterioscler Thromb Vasc Biol.

[8] Akboga MK, Balci KG, Maden O, Ertem AG, Kirbas O, Yayla C, Acar B, Aras D, Kisacik H, Aydogdu S (2016). Usefulness of monocyte to HDL-cholesterol ratio to predict high SYNTAX score in patients with stable coronary artery disease. Biomark Med.

[9] Kundi H, Kiziltunc E, Cetin M, Cicekcioglu H, Cetin ZG, Cicek G, Ornek E (2016). Association of monocyte/HDL-C ratio with SYNTAX scores in patients with stable coronary artery disease. Herz.

[10] Zhang Y, Li S, Guo YL, Wu NQ, Zhu CG, Gao Y, Xu RX, Dong Q, Liu G, Sun J, Li JJ (2016). Is monocyte to HDL ratio superior to monocyte count in predicting the cardiovascular outcomes: evidence from a large cohort of Chinese patients undergoing coronary angiography. Ann Med.

[11] Cetin MS, Ozcan Cetin EH, Kalender E, Aydin S, Topaloglu S, Kisacik HL, Temizhan A (2016). Monocyte to HDL cholesterol ratio predicts coronary artery disease severity and future major cardiovascular adverse events in acute coronary syndrome. Heart Lung Circ.

[12] Acikgoz N, Kurtoğlu E, Yagmur J, Kapicioglu Y, Cansel M, Ermis N (2017). Elevated monocyte to high-density lipoprotein cholesterol ratio and endothelial dysfunction in Behçet disease. Angiology.

[13] Atan D, Kundi FCS, Özcan KM, Dere H (2017). A new predictor for obstructive sleep apnea syndrome: monocyte to HDL ratio. Indian J Otolaryngol Head Neck Surg.

[14] Inonu Koseoglu H, Pazarli AC, Kanbay A, Demir O (2016). Monocyte count/HDL cholesterol ratio and cardiovascular disease in patients with obstructive sleep apnea syndrome. Clin Appl Thromb Hemost.

[15] You S, Zhong C, Zheng D, Xu J, Zhang X, Liu H, Zhang Y, Shi J, Huang Z, Cao Y, Liu CF (2017). Monocyte to HDL cholesterol ratio is associated with discharge and 3-month outcome in patients with acute intracerebral hemorrhage. J Neurol Sci.

[16] Baek K, Chung I (2017). Cadmium exposure is associated with monocyte count and monocyte to HDL ratio, a marker of inflammation and future cardiovascular disease in the male population. J Korean Med Sci.

[17] Cagli K, Tok D, Turak O, Gunertem E, Yayla C, Lafci G, Ulas MM, Cagli K (2016). Monocyte count-to-high-density lipoprotein-cholesterol ratio is associated with abdominal aortic aneurysm size. Biomark Med.

[18] Koçak HE, Acipayam H, Elbistanlı MS, Yiğider AP, Alakhras W, Kıral MN, Kayhan FT (2016). Is the monocyte/HDL ratio a prognostic marker of idiopathic sudden hearing loss?. Otolaryngol Pol.

[19] Kundi H, Gok M, Kiziltunc E, Cetin M, Cicekcioglu H, Cetin ZG, Karayigit O, Ornek E (2015). Relation between monocyte to high-density lipoprotein cholesterol ratio with presence and severity of isolated coronary artery ectasia. Am J Cardiol.

[20] Aydin E, Ates I, Fettah Arikan M, Yilmaz N, Dede F (2017). The ratio of monocyte frequency to HDL cholesterol level as a predictor of asymptomatic organ damage in patients with primary hypertension. Hypertens Res.

[21] Canpolat U, Çetin EH, Cetin S, Aydin S, Akboga MK, Yayla C, Turak O, Aras D, Aydogdu S (2016). Association of Monocyte-to-HDL cholesterol ratio with slow coronary flow is linked to systemic inflammation. Clin Appl Thromb Hemost.

[22] Dogan A, Oylumlu M (2017). Increased monocyte-to-HDL cholesterol ratio is related to cardiac syndrome X. Acta Cardiol.

[23] Vahit D, Mehmet KA, Samet Y, Hüseyin E (2017). Assessment of monocyte to high density lipoprotein cholesterol ratio and lymphocyte-to-monocyte ratio in patients with metabolic syndrome. Biomark Med.

[24] Wei Xue-biao, Chen Feng, Huang Jie-leng, He Peng-cheng, Wei Yan-xing, Tan Ning, Chen Ji-yan, Yu Dan-qing, Liu Yuan-hui (2018). Novel Risk Biomarker for Infective Endocarditis Patients With Normal Left Ventricular Ejection Fraction　― Monocyte to High-Density Lipoprotein Cholesterol Ratio ―. Circulation Journal.

[25] Akboga MK, Yayla C, Balci KG, Ozeke O, Maden O, Kisacik H, Temizhan A, Aydogdu S (2017). Relationship between serum albumin level and monocyte to high density lipoprotein cholesterol ratio with saphenous vein graft disease in coronary bypass. Thorac Cardiovasc Surg.

[26] Kanbay M, Solak Y, Unal HU, Kurt YG, Gok M, Cetinkaya H, Karaman M, Oguz Y, Eyileten T, Vural A, Covic A, Goldsmith D, Turak O, Yilmaz MI (2014). Monocyte count/HDL cholesterol ratio and cardiovascular events in patients with chronic kidney disease. Int Urol Nephrol.

[27] Canpolat U, Aytemir K, Yorgun H, Şahiner L, Kaya EB, Çay S, Topaloğlu S, Aras D, Oto A (2015). The role of preprocedural monocyte-to-high-density lipoprotein ratio in prediction of atrial fibrillation recurrence after cryoballoon-based catheter ablation. Europace.

[28] Saskin H, Serhan Ozcan K, Yilmaz S (2017). High preoperative monocyte count/high- density lipoprotein ratio is associated with postoperative atrial fibrillation and mortality in coronary artery bypass grafting. Interact Cardiovasc Thorac Surg.

[29] Sağ S, Yıldız A, Aydin Kaderli A, Gül BC, Bedir Ö, Ceğilli E, Özdemir B, Can FE, Aydınlar A (2017). Association of monocyte to HDL cholesterol level with contrast induced nephropathy in STEMI patients treated with primary PCI. Clin Chem Lab Med.

[30] Açıkgöz SK, Açıkgöz E, Şensoy B, Topal S, Aydoğdu S (2016). Monocyte to high-density lipoprotein cholesterol ratio is predictive of in-hospital and five-year mortality in ST-segment elevation myocardial infarction. Cardiol J.

[31] Çiçek G, Kundi H, Bozbay M, Yayla C, Uyarel H (2016). The relationship between admission monocyte HDL-C ratio with short-term and long-term mortality among STEMI patients treated with successful primary PCI. Coron Artery Dis.

[32] Karataş MB, Çanga Y, Özcan KS, İpek G, Güngör B, Onuk T, Durmuş G, Öz A, Karaca M, Bolca O (2016). Monocyte to high-density lipoprotein ratio as a new prognostic marker in patients with STEMI undergoing primary percutaneous coronary intervention. Am J Emerg Med.

[33] Arısoy A, Altunkaş F, Karaman K, Karayakalı M, Çelik A, Ceyhan K, Zorlu Ç (2017). Association of the Monocyte to HDL cholesterol ratio with Thrombus burden in patients with ST-segment elevation myocardial infarction. Clin Appl Thromb Hemost.

[34] Cetin EH, Cetin MS, Canpolat U, Aydin S, Topaloglu S, Aras D, Aydogdu S (2015). Monocyte/HDL-cholesterol ratio predicts the definite stent thrombosis after primary percutaneous coronary intervention for ST-segment elevation myocardial infarction. Biomark Med.

[35] Tok D, Turak O, Yayla Ç, Ozcan F, Tok D, Çağlı K (2016). Monocyte to HDL ratio in prediction of BMS restenosis in subjects with stable and unstable angina pectoris. Biomark Med.

[36] Ucar FM (2016). A potential marker of bare metal stent restenosis: monocyte count - to- HDL cholesterol ratio. BMC Cardiovasc Disord.

[37] Yilmaz S, Akboga MK, Sen F, Balcı KG, Aras D, Temizhan A, Aydogdu S (2016). Usefulness of the monocyte-to-high-density lipoprotein cholesterol ratio to predict bare metal stent restenosis. Biomark Med.

[38] Combadière C, Potteaux S, Rodero M, Simon T, Pezard A, Esposito B, Merval R, Proudfoot A, Tedgui A, Mallat Z (2008). Combined inhibition of CCL2, CX-3CR1, and CCR5 abrogates Ly6C(hi) and Ly6C(lo) monocytosis and almost abolishes atherosclerosis in hypercholesterolemic mice. Circulation.

[39] Ghattas A, Griffiths HR, Devitt A, Lip GY, Shantsila E (2013). Monocytes in coronary artery disease and atherosclerosis: where are we now?. J Am Coll Cardiol.

[40] Westerterp M, Gourion-Arsiquaud S, Murphy AJ, Shih A, Cremers S, Levine RL, Tall AR, Yvan-Charvet L (2012). Regulation of hematopoietic stem and pro- genitor cell mobilization by cholesterol efflux pathways. Cell Stem Cell.

[41] Ansell BJ, Navab M, Hama S, Kamranpour N, Fonarow G, Hough G, Rahmani S, Mottahedeh R, Dave R, Reddy ST, Fogelman AM (2003). Inflammatory/anti-inflammatory properties of high-density lipoprotein distinguish patients from control subjects better than high-density lipoprotein cholesterol levels and are favorably affected by simvastatin treatment. Circulation.

[42] Yvan-Charvet L, Pagler T, Gautier EL, Avagyan S, Siry RL, Han S, Welch CL, Wang N, Randolph GJ, Snoeck HW (2010). Tall AR.ATP-binding cassette transporters and HDL suppress hematopoietic stem cell proliferation. Science.

[43] Kuvin JT, Rämet ME, Patel AR, Pandian NG, Mendelsohn ME, Karas RH (2002). A novel mechanism for the beneficial vascular effects of high-density lipoprotein cholesterol: enhanced vasorelaxation and increased endothelial nitric oxide synthase expression. Am Heart J.

